# The Effect of Core Exercise Using Online Videoconferencing Platform and Offline-Based Intervention in Postpartum Woman with Diastasis Recti Abdominis

**DOI:** 10.3390/ijerph19127031

**Published:** 2022-06-08

**Authors:** Seohee Kim, Donghyun Yi, Jongeun Yim

**Affiliations:** 1 Department of Physical Therapy, Graduate School, Sahmyook University, Seoul 01795, Korea; physio.shkim@gmail.com; 2 Institute of Active Aging, Sahmyook University, Seoul 01795, Korea; yidonghyun89@daum.net; 3 Department of Physical Therapy, Sahmyook University, Seoul 01795, Korea

**Keywords:** postpartum, diastasis recti, exercise intervention, telehealth

## Abstract

To investigate the efficacy of exercise intervention using a real-time video conferencing platform (ZOOM) on inter-recti distance, abdominal muscle thickness, static trunk endurance, and maternal quality of life, 37 women with diastasis recti between six months and one year postpartum were randomly divided into the online (n = 19) and offline (n = 18) groups. The online group underwent 40-min trunk stabilization exercise sessions twice a week for six weeks, through a real-time video conference platform, while the offline group attended the same program in person. The inter-recti distance and muscle thickness between the abdominal muscles were measured by rehabilitation ultrasound imaging, the Torso endurance test was used to compare the static trunk endurance, and the maternal quality of life questionnaire (MAPP-QOL, score) was applied. Significant improvements were observed in the inter-recti distance between the rectus abdominis, abdominal muscle thickness, static trunk endurance, and maternal quality of life in both groups (*p* < 0.001); a more significant improvement was observed in the offline group. No significant differences were observed between groups except for the left rectus abdominis thickness and Psychological/Baby and Relational/Spouse-Partner subscale in the maternal quality of life index (*p* > 0.05). Exercise interventions delivered in a real-time videoconferencing platform are effective at improving the inter-recti distance, trunk stability, and quality of life in postpartum women and may be an alternate to face-to-face intervention.

## 1. Introduction

Diastasis recti abdominis (DRA) refers to the separation between the rectus abdominis along the linea alba due to increased abdominal internal pressure, which commonly occurs during and after pregnancy [1]. Boissonnault (1988) reported a 66% incidence of DRA during the third quarter of pregnancy, while Hannaford and Tozer (1985) reported an incidence of up to 100% [2]. Although the evidence that DRA itself causes lower back pain and pelvic-related diseases is unclear [3], in a previous study, patients with DRA diagnosis were investigated to determine the common symptoms that were present at the time of diagnosis. Lower back pain, pelvic floor dysfunction, pelvic pain, and urinary incontinence were the main ones reported. Moreover, women with DRA have a significantly higher prevalence of associated diseases than women without [4]. Furthermore, increased skin and abdominal structural instability causes negative body image in women, negatively affecting their self-esteem and mental health [5] and worsening the quality of life [6]. In some women, weakened abdominal walls persist even during the postpartum period, damaging their ability to perform activities of daily living (ADL) [7].

The abdominal walls make up part of the abdominal cavity and play an important role in maintaining the stability and posture of the body and pelvis through internal abdominal pressure, breathing, and abdominal organ support. These structures include deep muscles, such as the transverse abdominis (TrA), which provide tension on the thoracolumbar fascia by adhering between the segments of the spine. Tension is essential for optimal trunk function [8]. Additionally, surface muscles such as the rectus abdominis (RA), external oblique (EO), and internal oblique (IO) muscles attach to the pelvis and chest to balance external loads [9,10,11]. Therefore, weakening of the abdominal muscles due to pregnancy also affects the functioning of the abdominal wall function, altering muscle strength and trunk endurance [12,13]. Abdominal exercise is generally recommended as an important physical therapy intervention for women with DRA [14]. The performance of core stabilization exercises after childbirth allows an average improvement of 35% in the effects of DRA [2]; depression and quality of life of postpartum women also display significant improvements [15]. However, the postpartum period is a time when social pressure to prioritize the needs of the newborn over the personal needs of the mother is at its strongest [16]. Furthermore, despite the physical and mental advantages that can be obtained through exercise, postpartum women often experience difficulty participating in regular exercise due to conflicts of interest with the need to perform housework, parenting, and work [17].

Furthermore, the movement of postpartum women in public has become more restricted due to the global pandemic caused by COVID-19, initially declared on 11 March 2020, by the World Health Organization (WHO) [18]. As an alternative, medical services through remote rehabilitation have been suggested, which has translated to providing medical rehabilitation services through the use of digital information services and communication technology [19]. This type of rehabilitation entails the use of information and communication technology to deliver services to people outside clinics, leading to reduced treatment costs and time [20]. Moreover, it has been reported that telemedicine services can be practically implemented by patients, family members, and healthcare providers during the COVID-19 pandemic [21]. These have also been recognized as a suitable method to maintain fitness levels while avoiding possible infection by the coronavirus by performing safe, simple, and easy-to-implement exercises at home [22].

Medical services using real-time video conferencing programs have recently emerged as a means of supervising patient movements and preventing injuries during exercise sessions via the Internet [23]. This method of instruction has demonstrated a better effect than Internet-based exercises with only one-way communication [24] and is also linked to patient motivation insofar as patients can receive immediate feedback [25]. 

Therefore, the purpose of this study was to compare the effects of core stabilization exercises conducted via face-to-face methods, as well as trunk stabilization exercises conducted using real-time video conferencing programs (e.g., ZOOM) on women between six months and one year postpartum. We hypothesized that there is no significant difference of the effect between online and off-line based exercise for postpartum women with DRA.

## 2. Materials and Methods

### 2.1. Study Design

**Figure 1:**
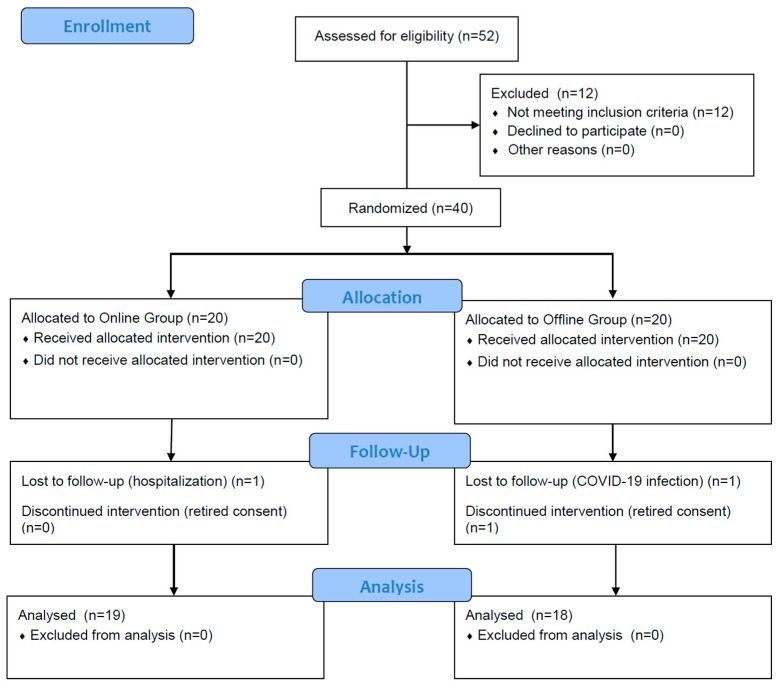
A flowchart of the study.

A two-group experimental design was used. The study compared pre-/post-test in each group and the difference between groups (Figure 1).

### 2.2. Participants

Fifty-two postpartum women at six months to one year following childbirth were recruited from online community between May 2020 and July 2020. Twelve of the fifty-two were excluded based on the criteria. Forty participants were randomly assigned to two groups via drawing lots. Three women dropped out, leaving thirty-seven women finally enrolled in the study. The criteria for selecting participants involved choosing those who had a two-finger distance or greater between the recti abdominis after self-examination using the two-finger method [26]. The measurements were implemented once more using ultrasound imaging (US) to exclude unsuitable participants by an experienced physical therapist prior to pre-testing. Further, those who understood the purpose of the study and participated in exercise sessions twice a week were included. The exclusion criteria were as follows: (1) participants with a history of chronic lower back pain and pelvic disease; (2) participants who were pregnant with more than one child during pregnancy (i.e., with twins or triplets); (3) participants with a history of chronic lower back pain and neurological signs such as numbness and tingling; (4) participants with a history of pelvic pain, urinary incontinence, and pelvic organ prolapse; (5) participants with cardiovascular and respiratory problems; (6) participants with scoliosis; (7) participants taking antipsychotic drugs; and (8) participants who had participated in regular exercise following childbirth. The general characteristics of the study participants are presented in Table 1.

### 2.3. Procedures

#### 2.3.1. Intervention Protocol

The core stabilization exercise program was constructed with reference to previous studies by Litos (2014) and Horsley (2020), and abided by the guidelines of the American College of Obstetricians and Gynecologists (ACOG); a rating of perceived exertion (RPE) of 15 was not exceeded to prevent excessive lactic acid production [27]. The Borg’s scale was used for RPE, which has been found to be effective in self-monitoring and self-regulation during exercise in a previous study by Carvalho et al. (2019). In addition, during each exercise session, the researcher provided a verbal reminder to the patient once so that the exercise intensity could be adjusted by the patient. In accordance with previous studies [28], the subjects recognized the spinal neutral posture through the “cat-camel posture” before the start of this exercise. In order to reduce the risk of errors and injuries due to inexperience with the exercise program, the hollowing technique, which requires three sets of lying down followed by pulling the belly button up for 10 s and finally by a period of relaxing, was implemented. During this time, researchers placed two fingers directly inside the patient’s anterior superior iliac spine (ASIS) to check if tension could be felt at the fingertips when the pelvic muscles contracted, and accurate core muscle contraction was induced for each attempt. Following the exercise program, the assigned weekly program was conducted with exercise movements performed according to the difficulty that each individual could perform. In this study, eccentric exercise was performed to activate type 2 muscle fibers [29]. 

During the six weeks of intervention, the two groups underwent the same core stabilization exercise program twice a week (12 sessions) during 40-min sessions. During the exercise program, the exercise sessions were conducted for the offline group at the same location the pre-test was held. For the experimental group, the exercise sessions were conducted via ZOOM on each individual’s home computer or device at a set time. In addition to regular sessions, it was recommended that the exercise programs be implemented as often as possible.

#### 2.3.2. Ultrasound Imaging

The inter-recti distance (IRD) and the thicknesses of the EO, IO, TrA, and RA were measured using an elliptical probe, B-mode, and 47–63 Hz of Rehabilitation ultrasound imaging (MySono U5, Samsung Medison, Seoul, Korea, 2010). The patient was placed in a supine position. All images were taken by maintaining the ultrasound probe horizontal to the abdomen without tilting it, and all measurements were taken with normal respiration to control for the effect of breathing and provide consistency among the participants [30]. When measuring the distance between both RA, landmarks were marked on the skin 2.5 cm above the top of the umbilicus with a nontoxic, aqueous marker while the participant was lying down, and a line was drawn horizontally with the linea alba. The thickness of the RA was measured by horizontally moving the line 2 cm above the umbilicus in the lateral direction [31], and the thicknesses of the EO, IO, and TrA were measured between the lower part of the thorax and iliac crest after placing the probe perpendicular to the transverse abdominal wall. A caliper of the ultrasound apparatus was used for measurement. Muscle thickness was measured by drawing a 2-cm line at the inner fascia end of each muscle and vertically crossing the previous line [32].

#### 2.3.3. Static Trunk Endurance Test

**Figure 2:**
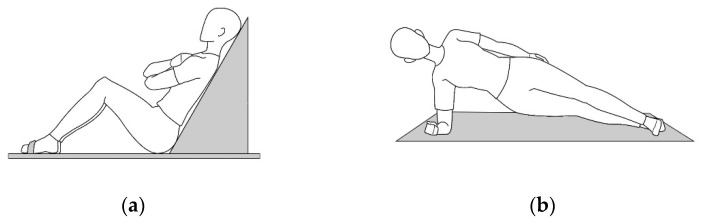
Static trunk endurance test: (**a**) Trunk flexor endurance test; (**b**) Bilateral side bridge test.

For endurance measurement, three core endurance tests were performed following the protocol of the Torso Muscular Endurance Test proposed by McGill et al. (1999): the trunk flexor endurance test and the bilateral side bridge tests. All movements were conducted after sufficient practice to eliminate any bias towards cognition, and the subjects aimed to maintain a static posture for as long as possible. The subjects sat with knees bent at 90° on the table for the trunk flexor endurance test, and their feet were held in place by a belt. Afterwards, they crossed their hands in front of their chests, leaned against the 60-degree trunk support, pulled the trunk support back 3 cm, and maintained this posture for as long as possible. The examination was halted when the subject’s leaning posture was lost or when their backs touched the support. The bilateral side bridge tests involved subjects lying on their side and raising their trunk with their knees straightened. The one foot was placed in front of the other, with hips raised, and they supported their weight with only the elbow. The examination was stopped when the posture of lying on their side was lost or when their buttocks fell to the mat (Figure 2).

#### 2.3.4. Maternal Quality of Life

The maternal perceived quality of life (MAPP-QOL) questionnaire was developed by Hill et al. (2006) and was based on Ferrans et al.’s (1998) Quality of Life Index (QLI). This questionnaire consists of thirty-eight items classified into five subcategories: Psychological/Baby (8 items), Socioeconomic (9 items), Relational/Spouse Partner (4 items), Relational/Family-Friends (9 items), and Health & Functioning (8 items). The internal consistency reliability (Cronbach’s alpha) of the maternal quality of life questionnaire was R = 0.96, and the test and retest reliability at two week intervals was R = 0.74.

### 2.4. Statistical Analysis

Statistical analysis was conducted by using SPSS software version 22.0 (SPSS Inc., Chicago, IL, USA). Shapiro–Wilk test was used for Normality test. The changes in IRD, Static trunk endurance and MAPP-QOL in the two groups were compared with the paired *t*-test. Difference between ZOOM groups and Control group were analyzed with the independent *t*-test. Statistical significance was set as *p ≤* 0.05.

## 3. Results

### 3.1. Participants Characteristics

The general characteristics of the subjects are shown in Table 1.

### 3.2. Inter-Recti Distance

The IRD showed a significant decrease from 1.99 ± 0.26 to 1.37 ± 0.40 in the online group and from 1.92 ± 0.30 to 1.18 ± 0.30 in the offline group following the sessions (*p* < 0.001); Moreover, there was no significant difference observed between the groups (*p* > 0.05) (Table 2).

### 3.3. Muscle Thickness

Table 3 shows the muscle thickness changes before and after the experiment, assessed using paired *t*-test. The thickness of the muscles showed a significant increase in both group, but there was a difference between groups only for the left rectus abdominis (*p* > 0.05).

### 3.4. Static Trunk Endurance

Table 4 displays static trunk endurance results. Both groups showed statistically significant increases after the experiment (*p* < 0.001), compared with those before. There was a significant difference in the endurance of the right side bridge tests between the groups (*p* < 0.05).

### 3.5. Maternal Quality of Life

Among the five subcategories of the MAP-QOL, the greatest improvement was observed in the health and functional areas (*p* < 0.001); Moreover, there was no significant difference between the two groups (*p* > 0.05). We observed a significant improvement in Online group (*p* < 0.001) in the spouse category, as compared with the offline group (*p* < 0.05) (Table 5).

## 4. Discussion

This study investigated the efficacy of conducting core stabilization movements over a period of six weeks through in person contact and real-time video conferencing contact at 6 months to 1 year postpartum in 37 female participants who were all diagnosed with DRA. We investigated the effect of IRD, abdominal muscle thickness, static trunk endurance, and maternal quality of life. We found that both groups showed a significant increase in IRD, static trunk endurance, and maternal quality of life, and there was no statistically significant difference when comparing between the two groups; however, a slightly significant improvement was demonstrated in the offline group.

After six weeks of intervention, IRD 2.5 cm above the umbilicus showed a statistically significant decrease in both groups, which is consistent with the results of previous study [33]. No significant difference was found between the two groups (*p* > 0.05) when comparing the differences in the intervention effects according to the exercise delivery method. In addition, for abdominal muscle thickness, a statistically significant increase in all abdominal muscles was observed after the intervention, indicating that the thickness of the muscle fibers increased noticeably through the stimulation of type 2 muscle fibers by the application of a program emphasizing eccentric exercise. No difference was identified between the two groups with respect to the thickness of the left rectus abdominis muscle. However, although there was no statistical difference between the groups, a slightly higher improvement rate was noted for IRD in the offline group. 

These changes in IRD and increased abdominal muscle thickness can lead to increased muscle strength and endurance [7]. In addition, static trunk stability following exercise also increased significantly. These findings are consistent with those of previous studies that demonstrated that core stabilization exercise contributes to trunk stabilization through muscle endurance improvement [34,35], there was no significant difference between the exercise delivery methods, except for a difference in the static endurance of the right trunk flexors. According to Carrick-Ranson et al., strengthening endurance through regular and continuous exercise has a positive effect on exercise ability, and making regular exercise a habit is important for future health care [36]. It has been said that it is necessary to create an appropriate environment for frequent exposure to exercise [37]; therefore, performing exercise via online media at home could contribute to improving health-related physical strength through regular exercise participation. 

The third outcome of this study was a change in overall maternal quality of life following the exercise delivery method. As stated in a previous review paper examining the correlation between exercise and depression, exercise can be an effective approach to treat depression in post-partum women, with outcomes equivalent to those noted after prescription drug use [38]. Therefore, this study measured the QOL of postpartum women in this study were assessed to compare how regular exercise intervention performed using different methods affects the overall QOL. Following the six-week exercise intervention, the health and functional areas, which are the subcategories with the lowest initial measurements, were the areas that were found to have shown the greatest improvements among both groups, indicating that exercise intervention significantly improves health and functional quality of life, with no significant differences observed between the delivery methods. In addition, it is noteworthy that the spouse and mental health areas showed significant differences between the two groups. Therefore, it could be inferred that during real-time video conferencing exercise sessions, the spouse took care of the children; thus, the emotional support improved the spouse’s relationship area and affected mental health-related areas [39,40]. Therefore, in future programs, it is suggested that organizing programs for families or groups to be eliminated restrictions on time and cost more effectively.

A unique feature of the use of real-time video conferencing sessions is that feedback can be provided more quickly, thus avoiding impairment due to insufficiency in delivery methods [41]. This is an important consideration in remote rehabilitation. Furthermore, this study had the advantage of easily recruiting participants, even when recruiting participants for the experimental group, because it was conducted through an online community of postpartum women. Moreover, in a previous study [42] that used physical therapy intervention and general physical therapy through remote rehabilitation to patients with heart failure, the quality of life related to health was significantly higher in the former than that in the latter general physical therapy group. In this study, it was found that all variables except the thickness of the left RA, mental health and child-related quality of life index, and spouse-related quality of life index were significantly improved but were slightly higher in the offline group. This suggests the need for an effective cueing method to narrow the gap between in person exercise interventions and online interventions, and in the future, the development of an online program conducted as a group through this cueing method may contribute to the health management of the community.

Thus, postpartum women lack personal time due to childcare and housework after childbirth, even if they are not in a COVID-19 situation, so it is of important clinical significance in that such tele exercise can relieve time-space constraints and save the cost of visiting clinics.

The limitations of this study are the difficulty of generalization due to the small number of participants and the differences that may appear as the intervention period increases due to the short intervention period. Furthermore, as all the subjects of this study were Asian, there were insufficient samples from other races. Therefore, in future studies, it will be necessary to increase the number of participants, as well as the duration of the intervention, to increase the effect of interim inspections during online interventions on the results. It will also be necessary to confirm changes in psychological variables, such as confidence in the ability to maintain dependent variables and perform exercises, through re-examination after a certain period of time. Moreover, it would be necessary to compare the differences using the same exercise program in other ethnicities.

## 5. Conclusions

In this study, we found that all variables except the thickness of the left RA, mental health and child-related quality of life index, and spouse-related quality of life index were slightly higher in the offline group than the experimental group. This suggests the need for an effective method to narrow the gap between face-to-face and online interventions. In the future, the development of an online program conducted as a group through this cueing method may additionally contribute to the health management of the community.

## Figures and Tables

**Table 1:** Participant characteristics (N = 37).

		Online Group (n = 19)	Offline Group (n = 18)	t (*p*)
Age	(year)	31.68 ± 3.92	32.72 ± 2.54	−0.951 (0.348)
Height	(cm)	163.19 ± 4.87	161.65 ± 6.85	0.791 (0.434)
Weight	(kg)	58.74 ± 6.80	59.56 ± 6.57	−0.370 (0.714)
BMI	(kg/m²)	21.97 ± 1.87	22.78 ± 1.95	−1.284 (0.208)
Birth Weight	(kg)	3.11 ± 0.43	3.07 ± 0.42	0.309 (0.759)
Delivery type				
Vaginal	N (%)	9 (24%)	6 (16%)	
Cesarean		10 (27%)	12 (32%)	

BMI, body mass index.

**Table 2:** Inter-recti distance (mm) (N = 37).

Location		Online Group (n = 19)	Offline Group (n = 18)	t (*p*)
2.5 cm above umbilicus	Pre-test	1.99 ± 0.26	1.92 ± 0.30	
	Post-test	1.37 ± 0.40	1.18 ± 0.30	
	Difference	−0.62 ± 0.27	−0.74 ± 0.23	1.471 (0.150)
	t (*p*)	10.143 (0.000)	13.745 (0.000)	

**Table 3:** Abdominal Thickness (cm) (N = 37).

		Online Group (n = 19)	Offline Group (n = 18)	t (*p*)
EO	Pre-test	0.33 ± 0.04	0.34 ± 0.04	
	Post-test	0.36 ± 0.04	0.38 ± 0.03	
	Difference	0.03 ± 0.02	0.04 ± 0.02	−0.637 (0.528)
	t (*p*)	6.245 (0.000)	8.030 (0.000)	
IO	Pre-test	0.54 ± 0.06	0.51 ± 0.05	
	Post-test	0.61 ± 0.07	0.60 ± 0.06	
	Difference	0.06 ± 0.03	0.08 ± 0.04	−1.295 (0.204)
	t (*p*)	8.182 (0.000)	8.517 (0.000)	
TrA	Pre-test	0.31 ± 0.02	0.33 ± 0.03	
	Post-test	0.35 ± 0.03	0.37 ± 0.03	
	Difference	0.04 ± 0.03	0.04 ± 0.02	−0.805 (0.426)
	t (*p*)	6.067 (0.000)	8.271 (0.000)	
RA (Left)	Pre-test	0.74 ± 0.06	0.77 ± 0.06	
	Post-test	0.84 ± 0.05	0.89 ± 0.07	
	Difference	0.08 ± 0.03	0.11 ± 0.05	−2.599 (0.014)
	t (*p*)	11.071 (0.000)	10.614 (0.000)	
RA (Right)	Pre-test	0.71 ± 0.07	0.76 ± 0.07	
	Post-test	0.83 ± 0.06	0.89 ± 0.06	
	Difference	0.11 ± 0.04	0.13 ± 0.04	−1.017 (0.316)
	t (*p*)	11.444 (0.000)	14.577 (0.000)	

EO, external oblique; IO, internal oblique; TrA, transversus abdominis; RA, rectus abdominis.

**Table 4:** Static trunk endurance (sec) (N = 37).

		Online Group (n = 19)	Offline Group (n = 18)	t (*p*)
Trunk Flexor	Pre-test	97.71 ± 35.05	106.08 ± 39.75	
	Post-test	113.40 ± 34.91	123.81 ± 37.69	
	Difference	15.69 ± 6.92	17.73 ± 8.86	−0.782 (0.439)
	t (*p*)	9.883 (0.000)	8.491 (0.000)	
Left Side Bridge	Pre-test	29.15 ± 12.11	32.00 ± 13.25	
	Post-test	34.83 ± 11.09	40.88 ± 12.49	
	Difference	5.68 ± 5.51	8.88 ± 5.50	−1.770 (0.085)
	t (*p*)	4.492 (0.001)	6.856 (0.000)	
Right Side Bridge	Pre-test	30.83 ± 10.92	34.38 ± 14.85	
	Post-test	37.35 ± 11.00	45.61 ± 13.68	
	Difference	6.52 ± 6.85	11.23 ± 5.63	−2.278 (0.029)
	t (*p*)	4.147 (0.001)	8.470 (0.000)	

**Table 5:** Changes in Maternal Quality of Life (points) (N = 37).

		Online Group (n = 19)	Offline Group (n = 18)	t (*p*)
Psychological/Baby	Pre-test	19.07 ± 2.68	17.80 ± 2.12	
	Post-test	22.74 ± 2.18	19.53 ± 2.46	
	Difference	3.66 ± 2.49	1.73 ± 2.43	2.359 (0.024)
	t (*p*)	6.253 (0.000)	3.031 (0.008)	
Socioeconomic	Pre-test	21.04 ± 3.91	20.16 ± 1.75	
	Post-test	22.71 ± 2.85	21.33 ± 1.97	
	Difference	1.67 ± 1.77	1.18 ± 1.72	0.844 (0.405)
	t (*p*)	3.996 (0.001)	2.902 (0.010)	
Relational/Spouse Partner	Pre-test	20.20 ± 3.59	20.10 ± 2.08	
	Post-test	23.16 ± 3.23	20.94 ± 3.31	
	Difference	2.96 ± 2.72	0.84 ± 3.14	2.163 (0.038)
	t (*p*)	4.610 (0.000)	1.136 (0.272)	
Relational/Family-Friends	Pre-test	19.20 ± 2.34	18.80 ± 2.57	
	Post-test	19.91 ± 2.25	19.76 ± 1.62	
	Difference	0.69 ± 2.03	0.96 ± 1.73	−0.416 (0.680)
	t (*p*)	1.445 (0.167)	2.343 (0.032)	
Health & Functioning	Pre-test	17.71 ± 2.34	17.94 ± 2.46	
	Post-test	20.79 ± 1.96	21.49 ± 1.77	
	Difference	3.08 ± 2.65	3.55 ± 1.82	−0.614 (0.544)
	t (*p*)	4.935 (0.000)	8.259 (0.000)	

## Data Availability

Not applicable.
